# Function of Transcription Factors *PoMYB12*, *PoMYB15*, and *PoMYB20* in Heat Stress and Growth of *Pleurotus ostreatus*

**DOI:** 10.3390/ijms241713559

**Published:** 2023-08-31

**Authors:** Hui Yuan, Zongqi Liu, Lifeng Guo, Ludan Hou, Junlong Meng, Mingchang Chang

**Affiliations:** 1 College of Food Science and Engineering, Shanxi Agricultural University, Jinzhong 030801, China; yuanhui201228@163.com (H.Y.); liuzongqisxau@126.com (Z.L.); mengjunlongseth@hotmail.com (J.M.); 2 Shanxi Engineering Research Center of Edible Fungi, Jinzhong 030801, China

**Keywords:** *Pleurotus ostreatus*, MYB transcription factor, heat stress, growth and development

## Abstract

MYB transcription factors (TFs) have been extensively studied in plant abiotic stress responses and growth and development. However, the role of MYB TFs in the heat stress response and growth and development of *Pleurotus ostreatus* remains unclear. To investigate the function of *PoMYB12*, *PoMYB15*, and *PoMYB20* TFs in *P. ostreatus*, mutant strains of *PoMYB12*, *PoMYB15*, and *PoMYB20* were generated using RNA interference (RNAi) and overexpression (OE) techniques. The results indicated that the mycelia of OE-*PoMYB12*, OE-*PoMYB20*, and RNAi-*PoMYB15* mutant strains exhibited positive effects under heat stress at 32 °C, 36 °C, and 40 °C. Compared to wild-type strains, the OE-*PoMYB12*, OE-*PoMYB20*, and RNAi-*PoMYB15* mutant strains promoted the growth and development of *P. ostreatus*. These mutant strains also facilitated the recovery of growth and development of *P. ostreatus* after 24 h of 36 °C heat stress. In conclusion, the expression of *PoMYB12* and *PoMYB20* supports the mycelium’s response to heat stress and enhances the growth and development of *P. ostreatus*, whereas *PoMYB15* produces the opposite effect.

## 1. Introduction

MYB transcription factors (TFs) are widely distributed in eukaryotes and feature highly conserved MYB binding domains that regulate diverse physiological processes in organisms [1]. In plants, the role of MYB TFs has been extensively studied, revealing their involvement in various abiotic stress responses and the growth and development of diverse species [2,3,4]. These abiotic stress responses primarily include drought stress, salt stress, cold stress, and heat stress. For instance, research on drought stress and salt stress has shown that rice’s OsFLP (MYB TF) plays a role in both drought stress and salt stress responses by regulating the expression of *OsNAC1* and *OsNAC6* [5]. The tobacco NtMYB102, analogous to *Arabidopsis thaliana*’s AtMYB70, shows increased tolerance to drought and salt stress upon overexpression (OE) of the coding gene [6]. In contrast, overexpressing *FtMYB22* in *Fagopyrum tataricum* decreased transgenic *A. thaliana*’s tolerance to both drought and salt stress [7]. Studies on cold stress and heat stress have found that three MYB TFs (SaMYB098, SaMYB015, and SaMYB068) in *Santalum album* significantly participate in the cold stress response [8]. Within the tea plant, CsMYB45, CsMYB46, and CsMYB105 are involved in jasmonic acid signal transduction under cold stress [9]. Moreover, OE of *OsMYB55* in rice enhances the amino acid metabolism of transgenic rice, thereby bolstering plant heat tolerance and mitigating the impact of high temperature on grain yield [10]. Meanwhile, *Lilium* LlMYB305 activates the *LlHSC70* promoter activity under heat stress, contributing to plant heat tolerance [11].

MYB TFs also assume significant roles in growth and development, as evidenced by their participation in seed germination, seedling development, root growth, leaf development, and anther development. For example, the MYB protein RSM1 in *A. thaliana* interacts with HY5/HYH to regulate seed germination and seedling development [12], while AtMYB103 can regulate anther development [13]. Notably, the LcMYB2 in *Leymus chinensis* promotes seed germination and root growth during drought stress [14]. Similarly, NbPHAN (MYB TF) promotes leaf development in *Nicotiana benthamiana* [15]. These investigations collectively underscore the potential involvement of MYB TFs in stress responses and growth and development across diverse plant species. However, the exploration of the engagement of MYB TFs in stress responses and growth development in fungi has been less comprehensive, with investigations limited to *Fusarium graminearum* and *Acremonium chrysogenum* [16,17]. Among these, the role of MYB TFs in abiotic stress response and growth and development in macro-fungi (edible fungi) has been relatively overlooked.

Edible fungi possess significant edible and medicinal values. Currently, research into their abiotic stress response mechanism and growth and development has emerged as a research hotspot. For example, the transcription factor FvHmg1 has been identified as a negative regulator of fruiting body development in *Flammulina velutiper* [18]. Similarly, the transcription factor PDD1 influences the development and yield of *F. velutiper* [19]. Furthermore, genes such as *hsp70*, *hsp90*, and *fes1* contribute to the regulation of heat stress during the initial stages of fruiting body development in *F. velutiper* [20]. Notably, within the phenol propane pathway, two *pal* genes were discovered to be implicated in fruiting body development and the mycelial heat stress response in *P. ostreatus* studies [21]. Additionally, the nitric-oxide-induced oxidase *aox* gene participates in regulating reactive oxygen species and enhancing resistance to heat stress in *P. ostreatus* [22]. In the realm of *Lentinus edodes*, research has revealed that the OE of the *hsp20* can enhance mycelial heat resistance [23]. In summation, numerous genes, including TFs, play an important role in the abiotic stress response and growth and development of edible fungi. However, there are few reports about MYB TFs.

*P. ostreatus* is among the most widely cultivated edible mushrooms globally, valued for its nutritional and medicinal attributes. In China, the cultivation of *P. ostreatus* predominantly occurs within horticultural facilities, enabling year-round production. Among these practices, the price of *P. ostreatus* produced in summer is the highest [24]. This pricing is largely due to the elevated summer temperatures, which foster the potential for mycelial scalding during its growth phase, consequently retarding mycelial development and causing mycelial demise. Mycelia play a pivotal role in the growth and maturation of *P. ostreatus*’s fruiting body. The formation of the fruiting body depends on the interlacing of the mycelium, with the mycelium serving as a vital nutrient repository for *P. ostreatus*, facilitating its growth and development. As such, robust mycelial growth forms the bedrock for ensuring optimal yields. Moreover, *P. ostreatus* boasts a short production cycle and notable conversion efficiency [25]. Enhancing its growth and development rate could shorten the production cycle and heighten yields, especially during the summer, with its high commodity prices. Such advancements hold substantial potential to propel the industrial progression of *P. ostreatus*. Consequently, in-depth investigations into *P. ostreatus*’s responses to heat stress and the mechanisms underlying its growth and development remain of utmost significance.

Prior transcriptome studies regarding *P. ostreatus*’s response to heat stress and its growth and development have revealed that *PoMYB12*, *PoMYB15*, and *PoMYB20* may be involved in heat stress response and growth and development [26]. Nevertheless, specific experiments to validate the functions of these TFs are lacking. In this study, RNA interference (RNAi) and OE techniques were used to verify the roles of these TFs in heat stress response and the growth and development of *P. ostreatus*. This work aims to provide a new theoretical basis for studying the heat stress response and growth and development mechanisms of *P. ostreatus*.

## 2. Results

### 2.1. PoMYB12, PoMYB15, and PoMYB20 May Be Involved in the Response of P. ostreatus Mycelium to Heat Stress

**Figure 1:**
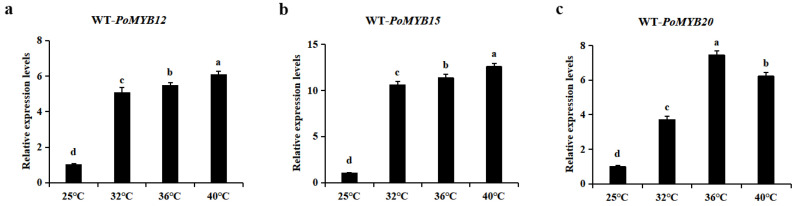
Detection of gene expression changes in WT strain mycelium at 32 °C, 36 °C, and 40 °C after 24 h of heat stress. (**a**) *PoMYB12* gene expression was detected in mycelia of WT strains. (**b**) *PoMYB15*. (**c**) *PoMYB20*. The expression levels of *PoMYB12*, *PoMYB15*, and *PoMYB20* in the mycelium of the WT strain without stress (25 °C) were used as controls and were set to 1. Each value represents the mean ± SD (*n* = 3). ANOVA and Duncan’s multiple range test (*p* < 0.05).

To investigate the alterations in gene expression of *PoMYB12*, *PoMYB15*, and *PoMYB20* in response to heat stress in *P. ostreatus* hyphae, mycelia from the WT were collected following 24 h of heat stress at 32 °C, 36 °C, and 40 °C. Specific primers were devised based on the nucleotide sequences of these three *MYB* genes, and they were cloned for *PoMYB12*, *PoMYB15*, and *PoMYB20*, respectively. Their open reading frames had total lengths of 1272 bp, 1440 bp, and 1947 bp, respectively (Supplementary Figure S1). Subsequently, the gene expression was assessed. The findings revealed variable degrees of upregulation in the expression of *PoMYB12*, *PoMYB15*, and *PoMYB20* under heat stress at 32 °C, 36 °C, and 40 °C. Notably, when the mycelium was subjected to heat stress at 32 °C to 40 °C, the expression level of *PoMYB12* increased by 5–6 times compared with the control, and the expression level was the highest at 40 °C (Figure 1a). *PoMYB15* also had the highest expression at 40 °C (Figure 1b). The highest expression level of *PoMYB20* occurred at 36 °C (Figure 1c). These results suggest that *PoMYB12*, *PoMYB15*, and *PoMYB20* may be involved in the heat stress response of *P. ostreatus* mycelia.

### 2.2. PoMYB12, PoMYB15, and PoMYB20 Are Involved in the Response of P. ostreatus Mycelium to Heat Stress

To verify the impacts of the *PoMYB12*, *PoMYB15*, and *PoMYB20* in *P. ostreatus* hyphae’s heat stress response, OE and RNAi experiments were conducted on these three *MYB* genes. The structural diagrams of the OE and RNAi plasmids for *PoMYB12*, *PoMYB15*, and *PoMYB20* are illustrated in Figure S2.

**Figure 2:**
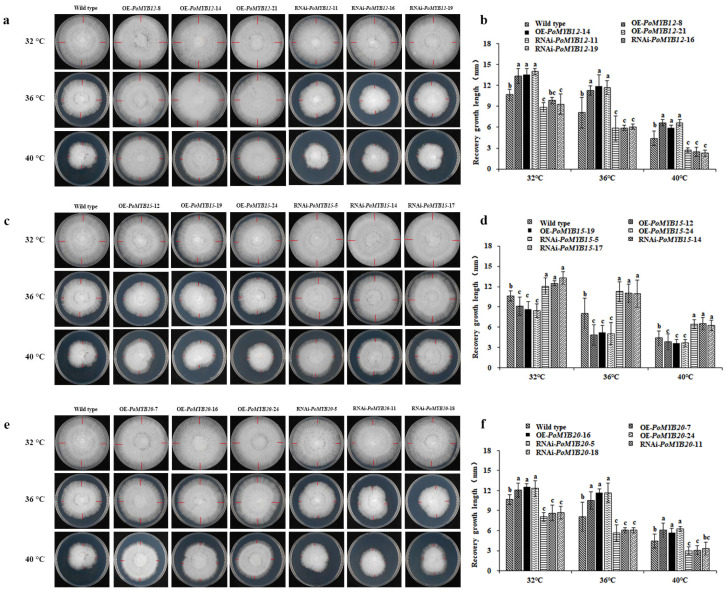
Observation of the recovery of colony morphology after 24 h of growth in mycelium of WT and mutant strains after 24 h of heat stress at 32 °C, 36 °C, and 40 °C. (**a**,**b**) Colony morphology and recovery of mycelial growth length of *PoMYB12* mutant strains. (**c**,**d**) *PoMYB15.* (**e**,**f**) *PoMYB20*. The WT strain was used as a control. The red line indicates the length of mycelium recovery growth.

Using the research method of Ludan Hou [27], the amplification of the hygromycin (Hyg) gene in *P. ostreatus*, transformed with the OE-*PoMYB12* plasmid, was conducted (Figure S3a). Subsequently, the relative expression level of the *PoMYB12* in the transformed strains was detected. The results showed that the expression levels of the *PoMYB12* in the OE-*PoMYB12*-8, OE-*PoMYB12*-14, and OE-*PoMYB12*-21 mutant strains were 4.74, 3.41, and 5.39 times that of the wild-type (WT) strain, respectively (Figure S3b). Similarly, the *Hyg* gene in *P. ostreatus*, transformed with the RNAi-*PoMYB12* plasmid, was amplified using the same methodology (Figure S3c). The results demonstrated that the expression levels of the *PoMYB12* in the RNAi-*PoMYB12*-11, RNAi-*PoMYB12*-16, and RNAi-*PoMYB12*-19 mutant strains were reduced by 70.1%, 61.7%, and 70.5%, respectively, compared with the WT strain (Figure 2b). Using the same method, OE mutant strains, namely OE-*PoMYB15*-12, OE-*PoMYB15*-19, and OE-*PoMYB15*-24 of *PoMYB15*, were obtained (Figure S3d,e). Concurrently, RNAi mutant strains, namely RNAi-*PoMYB15*-5, RNAi-*PoMYB15*-14, and RNAi-*PoMYB15*-17 of *PoMYB15*, were successfully generated (Figure S3e,f). Furthermore, OE mutant strains, namely OE-*PoMYB20*-7, OE-*PoMYB20*-16, and OE-*PoMYB20*-24 of *PoMYB20* were obtained (Figure S3g,h). The RNAi mutant strains RNAi-*PoMYB20*-5, RNAi-*PoMYB20*-11, and RNAi-*PoMYB20*-18 of *PoMYB20* were obtained (Figure S3h,i).

The WT strain, alongside OE and RNAi mutant strains of *PoMYB12*, *PoMYB15*, and *PoMYB20*, were cultured on potato dextrose agar (PDA) medium, cultured at 25 °C in the dark for 4 d, transferred to 32 °C, 36 °C, and 40 °C in the dark for 24 h, and then transferred to 25 °C in the dark to resume growth for 24 h. The WT strain was used as the control. The mycelia of OE strains of *PoMYB12* and *PoMYB20* had stronger recovery ability than the WT strain after 32 °C, 36 °C, and 40 °C heat stress, while RNAi strains were weaker than WT strains (Figure 2a,b,e,f). Interestingly, the mycelium recovery ability of OE strains of *PoMYB15* were weaker than that of the WT strain after 32 °C, 36 °C, and 40 °C heat stress, whereas the research results of RNAi strains were the opposite (Figure 2c,d).

### 2.3. PoMYB12, PoMYB15, and PoMYB20 May Be Involved in the Growth and Development of P. ostreatus

**Figure 3:**
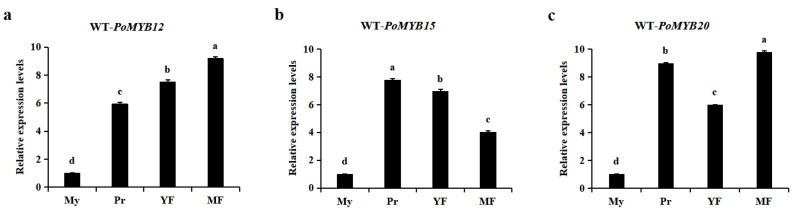
Changes in gene expression levels of *PoMYB12*, *PoMYB15*, and *PoMYB20* during the growth and development of WT strain. (**a**) *PoMYB12*. (**b**) *PoMYB15*. (**c**) *PoMYB20*. The expression levels of the *PoMYB12*, *PoMYB15*, and *PoMYB20* in the mycelium of the WT strain were used as controls and were set to 1. My: mycelium, Pr: primordium, YF: young fruiting body, MF: mature fruiting body. Each value represents the mean ± SD (*n* = 3). ANOVA and Duncan’s multiple range test (*p* < 0.05).

In order to investigate the variations in gene expression of the *PoMYB12*, *PoMYB15*, and *PoMYB20* within the WT strain during distinct stages of growth and development, samples were collected from the mycelium, primordium, young fruiting body, and mature fruiting body, respectively. These samples were subjected to gene expression analysis. The outcomes indicated a gradual increase in the expression level of *PoMYB12* throughout the growth and development phases, with the highest expression observed in the mature fruiting body stage (Figure 3a). The expression level of *PoMYB15* was highest in the primordium stage, and decreased in the young fruiting body and mature fruiting body stages (Figure 3b). *PoMYB20* showed high gene expression in the primordium and mature fruiting body stage (Figure 3c).

### 2.4. PoMYB12, PoMYB15, and PoMYB20 Are Involved in the Growth and Development of P. ostreatus

**Figure 4:**
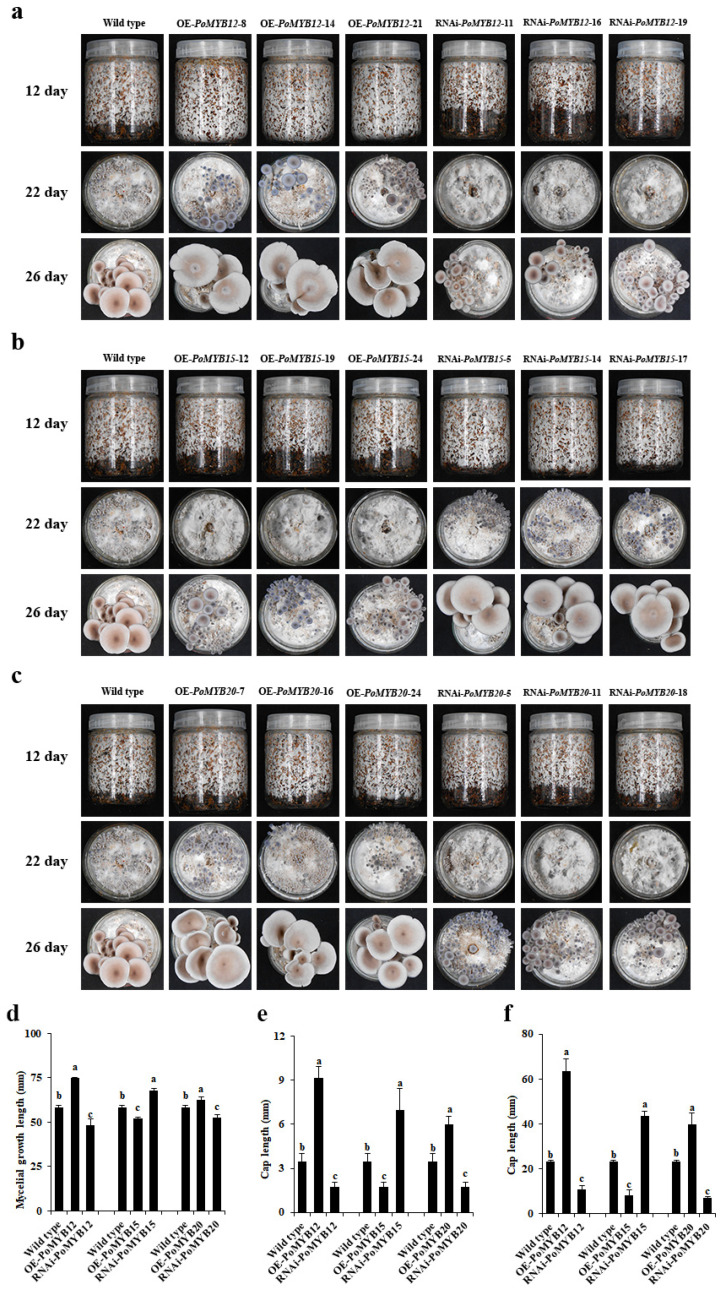
Growth and development of mutant strains. (**a**) Growth and development of *PoMYB12* mutant strains. (**b**) *PoMYB15* mutant strains. (**c**) *PoMYB15* mutant strains. (**d**) Mycelial growth length of mutant strains cultured for 12 d. (**e**) Cap length of mutant strains cultured for 22 d. (**f**) Cap length of mutant strains cultured for 26 d. WT strains were used as controls.

In order to investigate the contribution of *PoMYB12*, *PoMYB15*, and *PoMYB20* to the growth and development of *P. ostreatus*, the obtained OE and RNAi mutant strains were cultured and observed, with the WT strain serving as a reference. The results showed faster growth rates for mycelia, primordium, and fruiting bodies in OE strains of *PoMYB12* and *PoMYB20* compared to WT. In contrast, the RNAi strains displayed slower growth rates (Figure 4a,c–f). However, the mycelial growth, primordial growth, and fruiting body growth of the OE strains of *PoMYB15* were slower than those of the WT strain, whereas the results of the RNAi strains showed the reverse trend (Figure 4b,d–f). These findings underscore that the expression of *PoMYB12* and *PoMYB20* serves to facilitate the growth and development of *P. ostreatus*, whereas the expression of *PoMYB15* does not.

### 2.5. PoMYB12, PoMYB15, and PoMYB20 Are Involved in Growth and Development in Response to 36 °C Heat Stress of P. ostreatus

**Figure 5:**
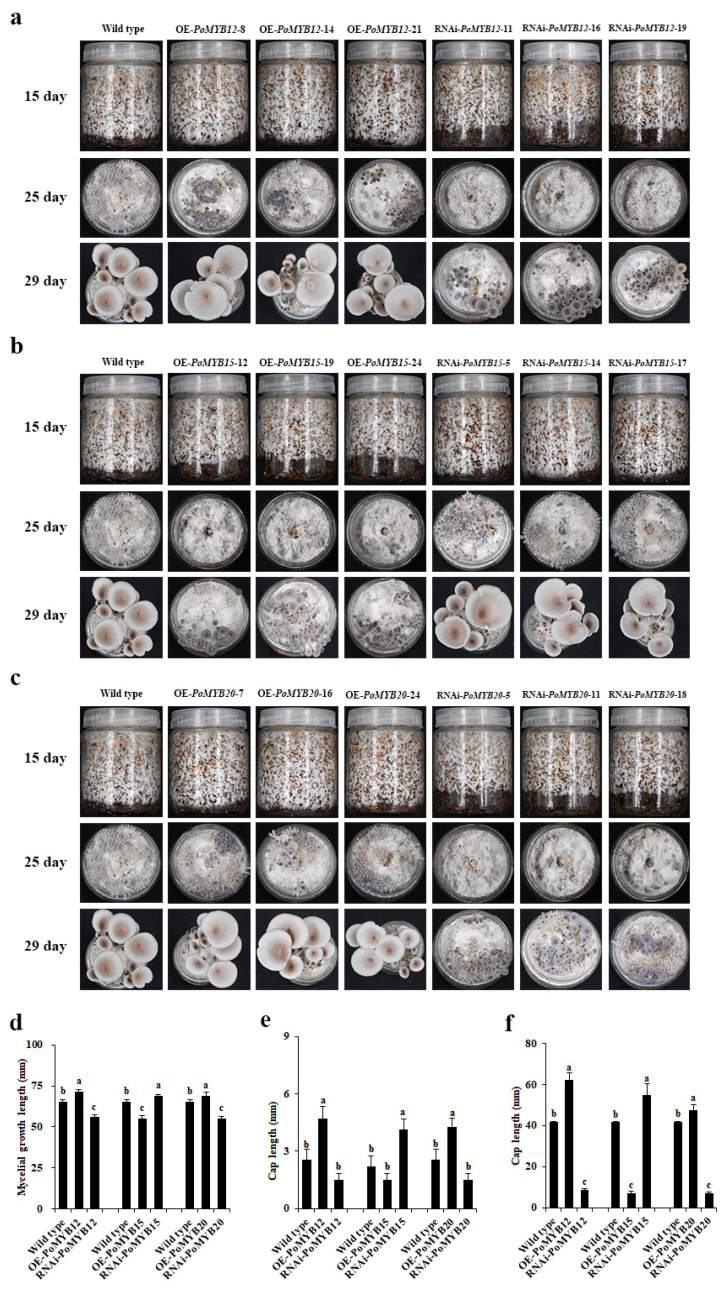
Observation of the recovery of mycelium growth and development of each mutant strain after 24 h of heat stress at 36 °C. (**a**) Growth and development results of *PoMYB12* mutant strains. (**b**) *PoMYB15* mutant strains. (**c**) *PoMYB20* mutant strains. (**d**) Mycelial growth length of mutant strains cultured for 15 d. (**e**) Cap length of mutant strains cultured for 25 d. (**f**) Cap length of mutant strains cultured for 29 d. The growth and development results of WT strain recovered after 24 h of 36 °C heat stress were taken as the control.

During the summer mycelial growth process, *P. ostreatus* frequently encounters brief episodes of high-temperature stress before resuming production. However, these brief instances of heat stress can lead to an elongation of the production cycle. To delve into the role of *PoMYB12*, *PoMYB15*, and *PoMYB20* in addressing high-temperature stress and participating in growth during mycelial development, combined with the research results of the mycelial heat stress response in this study, 36 °C was selected for heat stress treatment for 24 h. The growth and development of the *PoMYB12*, *PoMYB15*, and *PoMYB20* mutant strains were closely monitored. Results delineated that the mycelial recovery growth, primordial and fruiting body growth, and development of the OE strains of *PoMYB12* and *PoMYB20* were faster than those of the WT strain. Conversely, the RNAi strains displayed slower growth than those of the WT strain (Figure 5a,c–f). The mycelial recovery and primordial and fruiting body growth of the OE strains of *PoMYB15* were slower than those of the WT strain, while the RNAi strain showed opposite results (Figure 5b,d–f). In conclusion, *PoMYB12* and *PoMYB20* demonstrate an ability to expedite the recovery of growth and development in *P. ostreatus* following exposure to 36 °C heat stress, whereas *PoMYB15* does not.

## 3. Discussion

The involvement of MYB TFs in plant heat stress and growth, as well as development, has been extensively investigated [2,3,4]. Studies on heat stress and growth and development hold significant importance for the advancement of the *P. ostreatus* industry. This is because heat stress can disrupt the regular growth of *P. ostreatus* mycelia, subsequently affecting the pace of growth and development that, in turn, influences the overall growth cycle of *P. ostreatus*. Nevertheless, there have been limited inquiries into whether MYB TFs play a role in mycelial heat stress and growth and development in *P. ostreatus*. As such, the exploration of the involvement of MYB TFs in mycelial heat stress response and growth and development within *P. ostreatus* remains an especially crucial endeavor.

MYB TFs can participate in the heat stress response in *Pennisetum glaucum* [28], *Morus alba* [29], *Vaccinium corymbosum* [30], and rice [31], providing a foundation for exploring the potential role of MYB TFs in the heat stress response of *P. ostreatus*. In this study, *PoMYB12*, *PoMYB15*, and *PoMYB20* were selected for functional exploration based on the results of the previous identification of the *P. ostreatus MYB* gene family [26]. The results from the identification of the *MYB* gene family in *P. ostreatus* showed that the expression patterns of *PoMYB12*, *PoMYB15*, and *PoMYB20* under 40 °C heat stress varied somewhat from those in this study, which may be related to the duration of the heat stress. The predecessors treated their samples with heat stress for 48 h, whereas our samples were treated with heat stress for 24 h [26]. Despite these differences, both studies suggest that *PoMYB12*, *PoMYB15*, and *PoMYB20* may be involved in the response of *P. ostreatus* hyphae to heat stress. Further OE and RNAi experiment results showed that the mycelial recovery growth rate of OE-*PoMYB12*, RANi-*PoMYB15*, and OE-*PoMYB20* was faster than that of WT strain when subjected to heat stress at 32 °C, 36 °C, and 40 °C for 24 h. It is interesting that the results of the *MYB* gene family identification of *P. ostreatus* and the results of this study both found that *PoMYB15* was highly expressed under heat stress, whereas strains that overexpress *PoMYB15* showed delayed mycelial recovery and growth, suggesting that *PoMYB15* might be a transcriptional suppressor, and the expression of *PoMYB15* inhibited the expression of downstream target genes. It is not conducive to the heat stress response. The OE of *PoMYB12* and *PoMYB20* exhibits similar results to the RNAi of *PoMYB15*. This may be due to the fact that *PoMYB12* and *PoMYB20* may be transcriptional activators. In the study of the heat stress response in *P. ostreatus*, it was found that RNAi-*pal1* strains could reduce the sensitivity of mycelia to ROS, whereas RNAi-*pal2* strains responded to heat stress by reducing the sensitivity of mycelia to H_2_O_2_ [21]. The OE-*aox* strain can actively respond to 32 °C heat stress in mycelium [22]. After being subjected to heat stress, strain OE-*Mnsod1* has a faster mycelial recovery rate than the WT strain and participates in the mycelial heat stress reaction of *P. ostreatus* [27]. Therefore, determining whether *pal1*, *pal2*, *aox*, and *Mnsod1* are regulated by *PoMYB12*, *PoMYB15*, or *PoMYB20* and their involvement in the heat stress regulatory pathway warrants further investigation.

The role of MYB TFs in fungal growth and development, particularly in macro-fungi, is becoming the focus of research. In fungi, AfMybA, MYT1, and MYT2 have been found to be involved in growth and development [32,33,34]. Among macro-fungi, *F. velutiper* and *Ganoderma lucidum* MYB TFs were found to be involved in growth and development [35,36]. In the context of *P. ostreatus MYB* gene family identification, there has been speculation that *MYB* might play a role in growth and development [26]. These findings provide a reference for us to explore the possible involvement of *PoMYB12*, *PoMYB15*, and *PoMYB20* in the growth and development of *P. ostreatus*. The change trend of *PoMYB12*, *PoMYB15*, *PoMYB20* expression during the growth and development of the WT strain explored in this study is similar to that in the identification of the *MYB* gene family of *P. ostreatus* [26]. Further phenotypic observations of the OE and RNAi strains revealed that the OE-*PoMYB12*, RNAi-*PoMYB15*, and OE-*PoMYB20* strains promote the growth and development of *P. ostreatus* more than the WT strain. Interestingly, OE-*PoMYB12* and OE-*PoMYB20* exhibit similar effects in response to mycelial heat stress and growth and development. Whether there is possible interaction between *PoMYB12* and *PoMYB20*, or whether they play a synergistic role in growth and development, needs to be further explored. Additionally, OE-*PoMYB15* and OE-*PoMYB12*, and OE-*PoMYB20*, also showed opposite effects in the growth and development process, which promoted further speculation that *PoMYB15* may be a transcription suppressor. A higher gene expression level corresponds to greater inhibition of downstream target gene expression, and inhibited expression of *PoMYB15* results in accelerated mycelial growth and development. In the study of *P. ostreatus* growth and development, it was found that functional genes like *Mnsod1* [27], *LaeA*-like [37], *aco* [38], *Pofst3* [39], *Pofst4* [40], *pal1*, and *pal2* [21] can participate in the growth and development of *P. ostreatus*, but the transcriptional regulation of these functional genes is not clear. Whether they may be regulated by *PoMYB12*, *PoMYB15*, or *PoMYB20* to participate in the growth and development of *P. ostreatus* is a worthwhile avenue for exploration.

In the production of *P. ostreatus*, when high temperatures are encountered during the mycelium growth process, producers disperse the hyphal rods of mushrooms to cool them. Subsequently, the mycelium continues to grow and produce mushrooms. Therefore, while examining the heat stress response and growth and development mechanism of *P. ostreatus* mycelia separately, this study integrated these two aspects to better simulate the actual production process, which is different than previous studies [21,22]. The research results indicate that the mycelium recovery and development rate of OE-*PoMYB12*, RNAi-*PoMYB15*, and OE-*PoMYB20* strains is faster than that of WT strain after being subjected to 36 °C heat stress, which is consistent with the roles of *PoMYB12*, *PoMYB15*, and *PoMYB20* in heat stress and growth and development in this study. The interesting results obtained in this study reveal that *PoMYB12* and *PoMYB20* play similar roles. It is worth further exploration to determine whether there is a synergistic effect between them and if an interaction relationship exists between them. In addition, future work should consider identifying the key genes under the control of MYB and explaining their roles in bringing the observed phenotype, enhancing our capacity to delve deeper into gene functions and improving our regulatory pathway information.

In this study, OE and RNAi experiments were performed on *PoMYB12*, *PoMYB15*, and *PoMYB20*. The results showed that OE of *PoMYB12* and *PoMYB20*, along with RNAi of *PoMYB15*, improved recovery growth post mycelial heat stress, and could accelerate the growth and development of *P. ostreatus*. The results of this study are expected to provide an important reference basis for the breeding and technological improvement of new varieties of *P. ostreatus*, along with serving as a significant reference for in-depth research in the field of heat stress response and growth and development regulation of *P. ostreatus* and other macro-fungi.

## 4. Materials and Methods

### 4.1. Strains Tested

The *P. ostreatus* strain, “Da Ye 39”, was provided by the Shanxi Edible Fungi Germplasm Resource Collection Center of China. *Escherichia coli* DH5α was provided by Beijing Tsingke Biotechnology Co., Ltd. (Beijing, China). The *Agrobacterium tumefaciens* GV3101 strain was preserved in our laboratory.

### 4.2. Construction of OE and RNAi Plasmids of PoMYB12, PoMYB15, PoMYB20

The total RNA of mycelium was extracted using the TransZol Up kit (TransGen Biotech, Beijing, China). cDNA was synthesized by reverse transcription of total RNA using EasyScript^®^ One-Step gDNA Removal and cDNA Synthesis SuperMix Kits (TransGen Biotech, Beijing, China). The coding sequences (CDS) of the *PoMYB12*, *PoMYB15*, and *PoMYB20* genes were obtained from the NCBI database (GenBank: MH510323.1:55-1326; MH510318.1:1-1440; MH510311.1:1-1947). Primer Premier 5.0 was used to design *PoMYB12-*, *PoMYB15-*, and *PoMYB20*-specific primers (Table S1) to clone the *PoMYB12*, *PoMYB15*, and *PoMYB20* genes. The original OE plasmids kept in the laboratory were double digested with *Spe*I and *PspOM*I, and the cloned OE-*PoMYB12*, OE-*PoMYB15*, and OE-*PoMYB20* sequences (Table S1) were inserted into OE plasmids containing the *Hyg* resistance gene by homologous recombination to obtain OE-*PoMYB12*, OE-*PoMYB15*, and OE-*PoMYB20* recombinant plasmids, respectively [25,41]. The original RNAi plasmids preserved in the laboratory were double digested with *Spe*I and *Bgl*II, and the cloned RNAi-*PoMYB12-*Sense, RNAi-*PoMYB15-*Sense, and RNAi-*PoMYB20-*Sense sequences (Table S1) were inserted into the RNAi plasmids containing *Hyg* resistance genes by homologous recombination to obtain recombinant plasmids. Subsequently, the obtained recombinant plasmids were double digested with *Spe*I and *PspOM*I, and the cloned RNAi-*PoMYB12-*Anti, RNAi-*PoMYB15-*Anti, and RNAi-*PoMYB20-*Anti sequences (Table S1) were inserted into the recombinant plasmids using homologous recombination to obtain the RNAi-*PoMYB12*, RNAi-*PoMYB15*, and RNAi-*PoMYB20* recombinant plasmids [25,41].

### 4.3. Recombinant Plasmids Transformed into Competent Cells of A. tumefaciens

*A. tumefaciens* GV3101-competent cells were prepared according to the method in reference [42]. Recombinant plasmids of OE-*PoMYB12*, OE-*PoMYB15*, OE-*PoMYB20*, RNAi-*PoMYB12*, RNAi-*PoMYB15*, and RNAi-*PoMYB20* were added to the competent cells, mixed, and incubated on ice for 5 min, in liquid nitrogen for 1 min, and in a water bath at 37 °C for 5 min, respectively. Subsequently, 600 μL of nonresistant lysogeny broth (LB) medium was added and expanded at 28 °C for 3 h. Finally, the cells were coated into culture dishes containing kanamycin (kan) 50 μg/mL and rifampicin (rif) 20 μg/mL resistance and incubated upside down at 28 °C for 2 d. After picking spots, colonies were PCR and expanded. Subsequently, 600 μL of nonresistant LB medium was added, shaken at 28 °C for 3 h, applied to kan (50 μg/mL) and rif (20 μg/mL) in a resistant petri dish, and cultured upside down at 28 °C for 2 d. A single colony was selected for colony PCR, and the culture was expanded for standby.

### 4.4. A. tumefaciens-Mediated Recombinant Plasmid Transfer to the Mycelium of P. ostreatus

The mycelium of P. ostreatus was inoculated into PDA medium, cultured in the dark at 25 °C for 5 d, and then inoculated into complete yeast medium (CYM) by punching 0.5 cm blocks with a hole puncher, which were left at 28 °C and cultured in the dark for 2 d. A. tumefaciens containing the OE-*PoMYB12*, OE-*PoMYB15*, OE-*PoMYB20*, RNAi-*PoMYB12*, RNAi-*PoMYB15*, and RNAi-*PoMYB20* plasmids were expanded, centrifuged and collected, added to induction medium (IM) liquid medium, mixed, and induced for 5 h at 28 °C, 90 r/min and protected from light. The blocks in CYM were transferred into A. tumefaciens induced with OE-*PoMYB12*, OE-*PoMYB15*, OE-*PoMYB20*, RNAi-*PoMYB12*, RNAi-*PoMYB15*, and RNAi-*PoMYB20* plasmids and incubated at 28 °C for 5 h in the dark. Then, they were transferred to IM solid medium, covered with cellophane, and cultured in the dark at 28 °C for 3 d. They were transferred to CYM containing Hyg and cefotaxime (Cef) resistance for 30 d. The surviving mycelia were transferred into PDA medium and collected. DNA was extracted, and Hyg gene fragments were amplified. RNA was extracted from the strain with the amplified Hyg fragment and reverse transcribed into cDNA, and the expression levels of *PoMYB12*, *PoMYB15*, and *PoMYB20* were detected to obtain the transformed strain.

### 4.5. Detection of Gene Expression in WT Strain under Different Heat Stress Conditions

The mycelia of WT strain cultured at 25 °C in the dark for 4 d and subjected to 32 °C, 36 °C, and 40 °C heat stress for 24 h were collected. Then, RNA from mycelium was extracted and reverse transcribed into cDNA. The qPCR primers were designed using Primer Premier 5.0 for *PoMYB12*, *PoMYB15*, and *PoMYB20* (Table S1). The gene expression of *PoMYB12*, *PoMYB15*, and *PoMYB20* was detected in mycelium using ChamQ™ Uni-versal SYBR^®^ qPCR Master Mix (Vazyme, Nanjing, China) kits. The Bio-Rad CFX Connect TM Real-Time PCR System was used. Reaction system: 0.4 μL 10 μM upstream and downstream primers, 10 μL ChamQ Universal SYBR qPCR Master Mix (2×), 4 μL cDNA template, and 5.2 μL ddH_2_O. The reaction procedure was as follows: predenaturation at 95 °C for 30 s, then 40 cycles of 10 s at 95 °C and 30 s at 60 °C, and finally, extension at 72 °C for 30 s. *β-Actin* and *β-tubulin* were used as internal reference genes [22], and the relative expression was calculated using the 2^−ΔΔCt^ method with three biological replicates [43].

### 4.6. Detection of Heat Stress Resistance in PoMYB12, PoMYB15, and PoMYB20 Transformed Strains

To simulate mycelium scald encountered in the summer production of *P. ostreatus*, the obtained mutant strains were inoculated into PDA medium, cultured in the dark at 25 °C for 4 d, transferred to 32 °C, 36 °C, and 40 °C for 24 h, and then placed back in the dark at 25 °C for 24 h. The recovery of mycelia was observed. Mycelial recovery growth length was measured by the cross-cross method [44]. Measurement accuracy accurate to millimeters.

### 4.7. Analysis of Gene Expression Levels and Effects of Transformed Strains on the Growth and Development of P. ostreatus

To detect the gene expression levels of *PoMYB12*, *PoMYB15*, and *PoMYB20* during the growth and development of *P. ostreatus*, samples of mycelium, primordium, young fruiting body, and mature fruiting body were collected. RNA from samples was extracted and reverse transcribed into cDNA. The procedure for gene expression measurement was the same as that described in the material method above.

The obtained transformants were cultured in PDA medium for 7 d at 25 °C in the dark, respectively. Then, the seed blocks were inoculated into the culture bottle (each bottle was inoculated with 5 evenly sized seeds 1 cm in diameter). The cultivar formulation was 78% cotton seed hulls, 2% lime, 18% bran, 1% glucose, 1% gypsum, and 60% water content and incubated at 25 °C for 16 d in the dark. With the WT strain as the control, the mycelial growth rates of transformed strains were observed. Additionally, mushroom emergence experiments were conducted. During the catalytic primordium stage, the temperature was alternated at 15 °C and 26 °C for alternating temperature difference stimulation, with a humidity of 85%, and scattered light alternated with dark conditions. During the growth stage of the fruiting body, the temperature was controlled alternately between 23 °C and 26 °C, with a humidity of 85%, and scattered light alternated with dark conditions. Mycelial growth length and cap length were measured by the cross-cross method [44]. Among them, the measurement of cap length selects the largest cap in each mushroom culture bottle as the measurement standard. Measurement accuracy accurate to millimeters.

### 4.8. Growth and Development of Transformed Strains after 24 h of Response to 36 °C Heat Stress

The transformed strains were inoculated into cultivation flasks, respectively. After the mycelia were cultured for 10 d, they were transferred to dark conditions at 36 °C for 24 h of heat stress. Following the heat stress, the mycelium was transferred to dark conditions at 25 °C for further cultivation. The subsequent conditions for catalytic primordium and growth of fruiting bodies remained consistent with the parameters described in the materials and methods section above. The mycelial growth length and cap length measurement was the same as that described in the material method above.

### 4.9. Statistical Analysis

All analyzed data were biologically replicated at least three times. Data represent the mean ± standard deviation. IBM SPSS Statistics 26 software was used to determine significant differences. Analysis of variance (ANOVA) and Duncan’s multiple range test were performed to assess differences between samples.

## Data Availability

All the research results and data in this work have been included in the manuscript and Supplementary Materials. The original data involved in this study can be obtained from the first author or corresponding author through email upon reasonable request.

## Supplementary Materials

The supporting information can be downloaded at: https://www.mdpi.com/article/10.3390/ijms241713559/s1.
