# Supplementary material for: Function of Transcription Factors PoMYB12, PoMYB15, and PoMYB20 in Heat Stress and Growth of Pleurotus ostreatus
Source: Int J Mol Sci. 2023 Aug 31;24(17):13559. doi: 10.3390/ijms241713559 (PMC10487880; doi:10.3390/ijms241713559)
Supplement: Supplementary file 1 [file ijms-24-13559-s001.zip › ijms-2533541-supplementary.pdf]

Additional file

Table S1. Primers used in this study.

Primers	Sequence(5'→3')	Remark
Clone-*PoMYB12*-F	AAGTCCTCCGCTACCCCTGC	Gene cloning
Clone-*PoMYB12*-R	GCTCAGCTGGAGCTTGTTGCC	
Clone-*PoMYB15*-F	ATGGTGAACATACTTGGTCA	
Clone-*PoMYB15*-R	TGCGTTAGGCGGAAAAGACT	
Clone-*PoMYB20*-F	ATGACTGAAAGGAATGTCGG	
Clone-*PoMYB20*-R	CTTCGCTGATTGTGCTGGA	
RNAi-*PoMYB12*-Sense-F	CTATTATAAGACTAGTAAGCTGCTGCACTCA	Construction of RNAi plasmid
RNAi-*PoMYB12*-Sense-R	CGATGATTGTAGATCTGGCCGTCGCCAGTGG	
RNAi-*PoMYB12*-Anti-F	CTATTATAAGACTAGTTATCTGCATCCGTGA	
RNAi-*PoMYB12*-Anti-R	CAATTCTAGAGGGCCCAAGCTGCTGCACTCA	
RNAi-*PoMYB15*-Sense-F	CTATTATAAGACTAGTTCAAGTTCTTCATTCA	
RNAi-*PoMYB15*-Sense-R	CGATGATTGTAGATCTTCCATTGGCAAAGACT	
RNAi-*PoMYB15*-Anti-F	CTATTATAAGACTAGTCACCGGGCCGCCGTC	
RNAi-*PoMYB15*-Anti-R	CAATTCTAGAGGGCCCTTCAAAAGGATCTGC	
RNAi-*PoMYB20*-Sense-F	CTATTATAAGACTAGTGAAAACGACAACTGG	
RNAi-*PoMYB20*-Sense-R	CGATGATTGTAGATCTACACTGGAGGGAATT	
RNAi-*PoMYB20*-Anti-F	CTATTATAAGACTAGTACAAACTGAGTCCCA	
RNAi-*PoMYB20*-Anti-R	CAATTCTAGAGGGCCCGAAAACGACAACTGG	
OE-*PoMYB12*-F	GGTCAAAGTTACTAGTAAGTCCTCCGCTACC	Construction of OE plasmid
OE-*PoMYB12*-R	CAATTCTAGAGGGCCCGCTCAGCTGGAGCTT	
OE-*PoMYB15*-F	GGTCAAAGTTACTAGTATGGTGAACATACTT	
OE-*PoMYB15*-R	CAATTCTAGAGGGCCCTGCGTTAGGCGGAA	
OE-*PoMYB20*-F	GGTCAAAGTTACTAGTATGACTGAAAGGAAT	
OE-*PoMYB20*-R	CAATTCTAGAGGGCCCCTTCGCTGATTGTGC	
Hyg-F	TATTCCTTTGCCCTCGGACG	Detection of transformants
Hyg-R	ATGAAAAAGCCTGAACTCACC	
qPCR-*PoMYB12*-F	CTACTGCTGCTGGAGGGTG	Gene expression analysis
qPCR-*PoMYB12*-R	CCAAGAATTTGCGGATACA	
qPCR-*PoMYB15*-F	ATTTCGTCCCTGTTCCTG	
qPCR-*PoMYB15*-R	ATCCTGATAGCCCACTCG	
qPCR-*PoMYB20*-F	ATGTGACGGACCCAGAAC	
qPCR-*PoMYB20*-R	CGTAGACCGCTGAGTTGA	
β-tubulin-F	AGGCTTTCTTGCATTGGTACACGC	Endogenous control
β-tubulin-R	TATTCGCCTTCTTCCTCATCGGCA	
β-actin-F	GCGATGAACAATAGCAGGG	
β-actin-R	GCTGGTATCCACGAGACAAC	

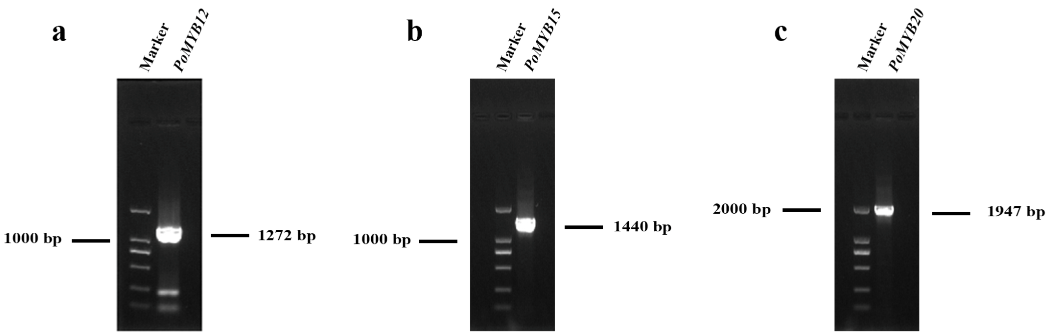

Figure S1. Cloning of *PoMYB12*, *PoMYB15*, and *PoMYB20* genes. (**a**). Cloning of *PoMYB12*. (**b**). Cloning of *PoMYB15*. (**c**). Cloning of *PoMYB20*.

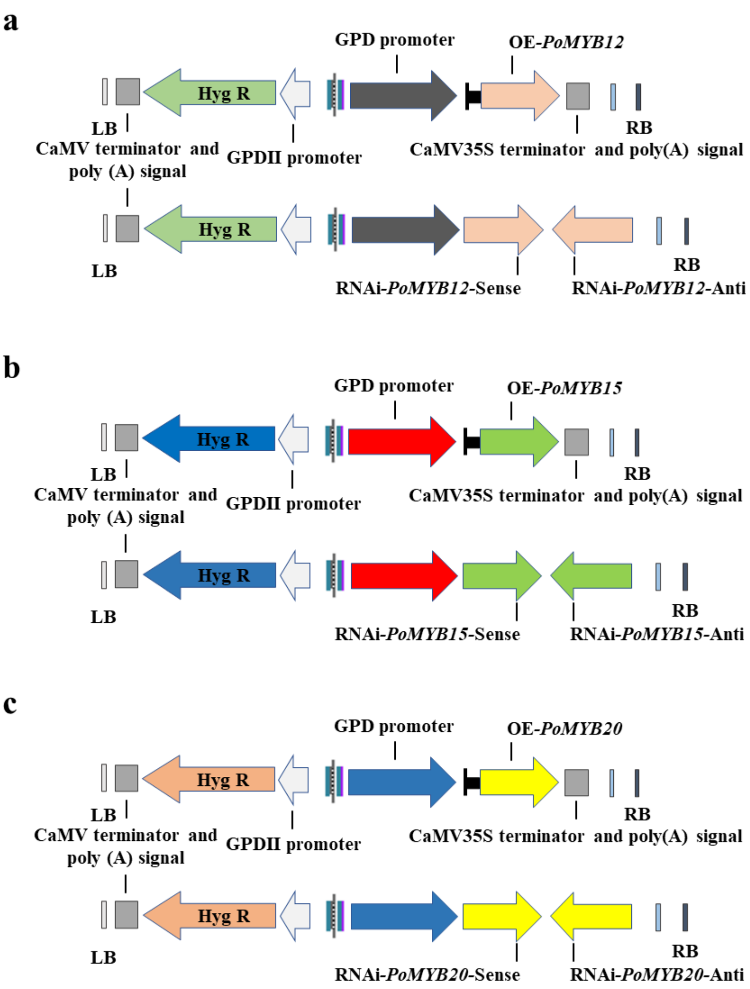

Figure S2. Schematic diagram of *PoMYB12*, *PoMYB15*, and *PoMYB20* OE and RNAi plasmid structure. (**a**). OE-*PoMYB12* and RNAi-*PoMYB12*. (**b**). OE-*PoMYB15* and RNAi-*PoMYB15*. (**c**). OE-*PoMYB20* and RNAi-*PoMYB20*.

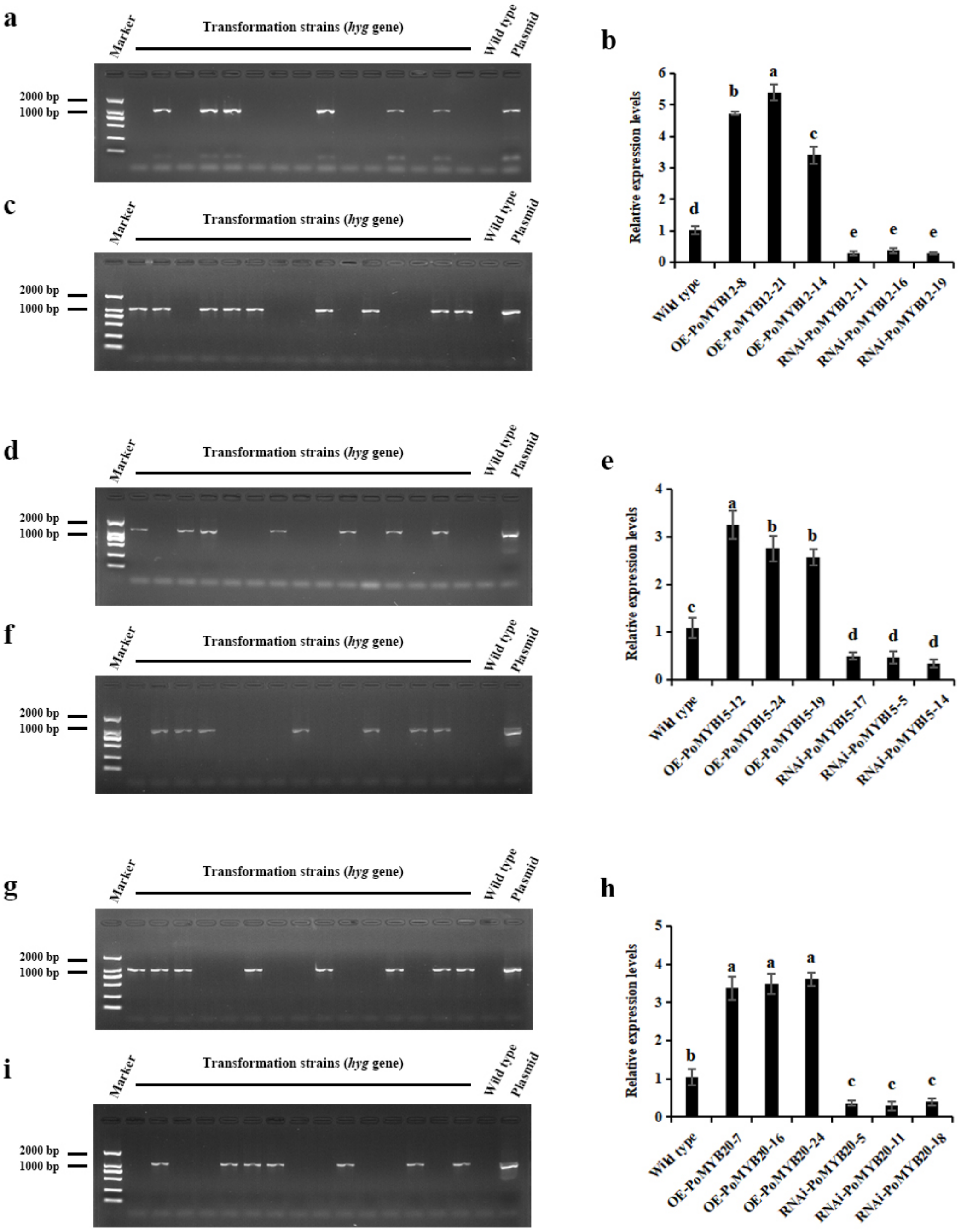

Figure S3. Screening of transforming strains. (**a**,**c**). Amplification of the *Hyg* gene in OE-*PoMYB12* and RNAi-*PoMYB12* mutant strains. (**b**). Analysis of *PoMYB12* gene expression level. (**d**,**f**). Amplification of the *Hyg* gene in OE-*PoMYB15* and RNAi-*PoMYB15* mutant strains. (**e**). Analysis of *PoMYB15* gene expression level. (**g**,**i**). Amplification of the *Hyg* gene in OE-*PoMYB20* and RNAi-*PoMYB20* mutant strains. (**h**). Analysis of *PoMYB20* gene expression level. The gene expression levels of *PoMYB12*, *PoMYB15* and *PoMYB20* in WT strains were set to 1. Each value represents the mean ± SD ($n = 3$). ANOVA and Duncan's multiple range test ($p < 0.05$).
